# Consumer objective and subjective knowledge about healthy foods: An approach to promote healthy lifestyle choices in South Africa

**DOI:** 10.1371/journal.pone.0296504

**Published:** 2024-01-25

**Authors:** Daleen van der Merwe, Hanli de Beer, Susanna Ellis, Petra Bester, Frederick Marais, Adri Steyn

**Affiliations:** 1 Faculty of Health Sciences, Africa Unit for Transdisciplinary Health Research, North-West University, Potchefstroom, South Africa; 2 Unit for Business Mathematics and Informatics, North-West University, Potchefstroom, South Africa; United Arab Emirates University, UNITED ARAB EMIRATES

## Abstract

Unhealthy food choices and consumption, coupled with sedentary lifestyles among consumers, intensify public health concerns regarding the quadruple disease burden, despite Primary Health Care (PHC) policy. However, the current relationship between consumer knowledge about healthy foods and following a healthy lifestyle needs to be explored. Our study, therefore, aimed to determine the association between consumers’ subjective and objective knowledge about healthy foods and various healthy lifestyle choices. A cross-sectional survey was conducted among employed consumers (*N* = 157) from South African corporate settings. We used structural equation modelling (SEM) to determine associations between subjective and objective knowledge about healthy foods and healthy lifestyle choices. Our findings showed that most participants scored high on making healthy lifestyle choices relating to avoiding smoking (69.5%) and limiting drinking alcohol (68.7%) but less so for food and sleep (44.4%) while neglecting exercise, relaxation (13.7%), and choices that require dedicated effort (25.2%). On average, participants had high levels of subjective (mean = 3.59; 5-point Likert scale) knowledge and objective knowledge about healthy foods (88.4−95.9% correct responses). However, their objective knowledge about weight and cholesterol had severe deficiencies (36.7%). SEM confirmed an association between subjective knowledge and most healthy lifestyle choice categories, while income contributed to dedicated effort lifestyle choices. By contrast, objective knowledge did not associate with such choices. Our structural model suggests that subjective knowledge about healthy foods contributes to healthy lifestyle choices. Therefore, subjective knowledge and the objective knowledge deficiencies we identified among corporate consumers can serve as a valuable starting point for informed education to promote PHC policy and healthy lifestyle choices.

## Introduction

Over the past four decades, consumer health and lifestyles have attracted much multidisciplinary research. Increasing awareness of healthy eating’s significant implications for well-being [1] and consumer interest in more nutritious food [2], combined with physical activity and fitness as a healthy lifestyle of choice, has gained momentum [3]. However, the disturbing prevalence of non-communicable diseases (NCDs) continues to rise, amounting to 41 million or 74% of global deaths in 2019 [4]. Unhealthy food and lifestyle choices are modifiable behavioural risk factors for NCDs and early death [4]. The negative implications of overweight and obesity resulting from overconsumption lead to harmful health conditions such as diabetes and cardiovascular complications [5,6]. Co-morbidities and high body mass index in COVID-19 patients are associated with more complications [7], with obesity posing a fivefold risk increase in mortality and extended hospital stays among these patients [8].

Statistical evidence underpins the importance of healthy lifestyle choices to improve South Africans’ general health and has underscored concerns about ongoing health-related challenges [9]. Of South Africa’s quadruple disease burden (i.e., a combination of NCDs, communicable diseases including HIV and tuberculosis, mother and child morbidity and mortality, violence and injuries exacerbated by poverty) [10], cardiovascular diseases exceed the burden of the remaining three [11]. South Africa has higher obesity rates in the developing world than the rest of Africa [12], which are still increasing [13]. Diabetes mellitus presents an increasing burden nationally, accounting for 5.7% of deaths in 2016 and rising from the seventh to the second largest cause of death between 2013 and 2016 [9]. NCDs in 2016 amounted to an estimated 50% of mortalities in this country [14]. Therefore, it is crucial to modify the behavioural risk factors for NCDs as far as possible by enabling healthy, active living through health education and promotion.

The implementation of several policies attempts to address the prominent health issues in South Africa. Public health policies such as Primary Care 101 Guideline 2013/14 (PC 101)–a symptom-based integrated clinical management approach to adult primary care [15]; the Integrated Chronic Disease Management (ICDM) model of care–a managed care model for integrated prevention, treatment and care of chronic disease on a primary health care level [16]; the Hospital Level (Adults) Standard Treatment Guidelines and Essential Medicine List–standardised treatment guidelines to ensure equitable access to safe, effective and affordable treatment of diseases [17] informs the comprehensive management also of lifestyle diseases in South Africa. Additionally, restrictive guidelines for salt in processed foods [18] and the sugar content of beverages apply [19]. The recently published draft Regulations for Labelling and Advertising of Foodstuffs (R.3337 of 2023) [20] is also foreseen to change the food labelling scene for consumers and the food industry in South Africa. A South African setting is thus an ideal starting point for investigating the relationship between consumer objective and subjective knowledge about healthy foods and their associated health-related lifestyle behaviours.

Already in 1972, Belloc and Breslow [21] advocated seven well-known lifestyle choices guiding the maintenance of good health: non-smoking, moderate-intensity physical activity, moderate to no alcohol consumption, seven to eight hours of sleep per night, body weight maintenance, eating breakfast daily, and regularly eating three meals per day. More recent research adds to the importance of any movement [22] and leisure-time physical activity, regardless of work-related physical activity [23], in health protection. Research has also illustrated the direct effect of maintaining healthy lifestyles on healthy ageing and emphasized the importance of accurate health messages and integrating routine behaviours into lifestyles [24]. These recommendations are illustrated by health-aware consumers primarily using food labels’ nutritional content and ingredient information to guide their ultimately healthier food choices [25]. Another study revealed that family members who witnessed diabetes complications were more attentive to their health and lifestyle modifications [26]. However, the World Health Organization [10] warns that adopting healthy lifestyles remains challenging despite broader awareness of the harmful consequences of poor food choices.

Healthy food choices accompanying healthy lifestyle habits should fight the negative impact of rising NCD rates, but food consumption is complex. Prinsloo et al. [27] advise that viewing food purchase decisions as a straightforward, rational process is probably an oversimplification due to consumers’ exposure to an overabundance of products and marketing-related information. Of these, price was of primary importance during food decisions regardless of nutrition or food quality in a South African study [28]. Hence, consumer decisions, whether about food or a healthy lifestyle, are complex and influenced by various marketing and social factors from consumers’ external environment and psychological factors from the internal environment [3]. However, the present study only focuses on subjective and objective knowledge and how they may relate to healthy lifestyle choices.

Consumers have been shown to rely on objective factual knowledge and their own subjective or ‘self-perceived’ knowledge when they make decisions [29–31]. The literature reveals that research on the correlations between objective and subjective knowledge categories and consumer health-related behaviours is inconclusive and may be category-specific. Objective and subjective knowledge affected health information seeking and the sources consulted differently [32]. The ongoing debate suggests a need for further research into the state of consumer subjective and objective knowledge about healthy foods and the connection, if any, of such knowledge and its role in encouraging the active pursuit of healthy living. The present study explores this connection by assessing consumers’ everyday healthy lifestyle choices. Our study aimed to clarify the value of consumer knowledge about health-related issues as a feature of encouraging healthy lifestyle choices. We drew on the concepts of subjective and objective knowledge about healthy foods—a vital component in healthy food choices—intending to establish their relationship with consumer decisions to pursue healthy lifestyles. Identifying and understanding these relationships would contribute valuable consumer information to the current health science literature on healthy lifestyle choices. This study is novel in terms of its specific African setting. Our sample comprised consumers in a corporate environment that elicits features that could be echoed in corporate structures in other countries.

We based our study on the following research questions:

1. To what extent did consumers adhere to various healthy lifestyle choices in South Africa?
2. What were these consumers’ subjective and objective knowledge about healthy foods?
3. What objective and subjective categories of knowledge about healthy foods were associated with healthy lifestyle choices, and to what extent did this knowledge about healthy foods contribute to these choices?

### Objective and subjective knowledge

Subjective knowledge is concerned with what consumers perceive they know as distinct from objective knowledge, which is the actual knowledge that is accurately stored in consumers’ long-term memory [29,33]. Therefore, for our study, ‘subjective knowledge’ refers to what and how much consumers think or perceive that they know about healthy foods, and ‘objective knowledge’ refers to the amount of accurate actual information about such foods they can recall from information stored in their memory.

Research has shown that the relationship between subjective and objective knowledge can be inconsistent [30,34]. Studies regarding various consumer topics revealed either positive relationships between subjective and objective knowledge [35,36] or discrepancies between the levels of these two types of knowledge [29,35]. These discrepancies result from individuals over- or under-estimating their factual knowledge. They are revealed when people’s levels of subjective knowledge are, respectively, higher or lower than their objective knowledge.

Research on subjective and objective knowledge has focused on establishing their relationship in the context of consumer behaviour and decision-making. As early as 1985, Brucks [29] proposed that subjective and objective knowledge influenced information processing in different ways. Subsequently, the power of subjective knowledge as a driver of behaviour has been highlighted. Some researchers have reported that subjective knowledge played a significant part in the greater consumption of organic vegetables [37] and fish [38], as well as increased food label usage [39] and information seeking on the human papillomavirus [32] and COVID-19 [40]. Raju et al. [36] argue that higher levels of subjective knowledge can result in greater certainty and confidence about the outcomes of decisions.

Regarding objective knowledge, studies have revealed correlations between objective nutrition knowledge and frequent nutritional label reading [41] and the role of this knowledge in mediating consumer attitudes and purchase intentions based on harmful nutrients and disease risks [42]. However, objective knowledge was only indirectly associated with organic vegetables [37] and fish [38] consumption. Also, consumers were more likely to change their behaviour with an increase in their subjective knowledge rather than with an increase in their objective knowledge [38]. However, objective knowledge was related to information searching based on more product attributes than subjective knowledge [29]. Hence, Brucks [29] emphasized that, despite the ease of searching based on subjective knowledge during decision-making, the outcome of this search is not necessarily founded on fact. On the other hand, objective knowledge alone might not be sufficient to increase label usage by consumers unless accompanied by subjective knowledge in which they see or perceive themselves as knowledgeable [39]. Thus, when high levels of objective knowledge accompany high levels of subjective knowledge, there is a sturdy basis for sound decision-making. For this reason, our study investigated both subjective and objective knowledge about healthy food in terms of their relationship to the active practising of health-related behaviours.

## Method

### Study setting

We undertook this study at three head offices of a single corporate corporation in three prominent city centres across South Africa: Pretoria, Durban, and Cape Town. This setting served our study’s purpose because this company offers employees a voluntary wellness programme. Corporate employee consumers can be particularly vulnerable to NCDs in mid-life or older, requiring monitoring and intervention [43].

### Study design and sampling

This study is part of a larger research project on healthy consumer lifestyle choices, and the parts of the questionnaire applicable to this study are provided in S1 Appendix. We conducted a cross-sectional online survey among employed consumers (n = 157) within the corporation willing to participate. Thus, it is not a random sample but an availability sample. Participating employees of the organization had to be 18 years or older and with internet access to complete the web-based questionnaire from July to October 2015. Convenience sampling was used per the inclusion criteria. Before and during the data collection, poster advertisements were placed in designated areas in these head offices to inform employees about the ongoing study. Emails were sent to employees to inform them about the study, obtain informed consent, and provide the link to the online questionnaire via QuestionPro. Using this platform allowed for anonymous participation. Willing participants could click on the link to the informed consent form and questionnaire without providing any personal information that could be linked to a specific employee.

### Data collection

The item compilation of the different constructs in the questionnaire is outlined in Table 1. Healthy lifestyle choices were measured using an adapted version of Belloc and Breslow’s [21] 7-item lifestyle habit index questionnaire. The original dichotomous scale (“yes”/”no” responses) was replaced with a 5-point Likert scale (1 = never to 5 = always) to determine the frequency with which participants followed specific healthy lifestyle choices. This scale was supplemented with nine related questions from the Lifestyle Profile II questionnaire [44]. Subjective knowledge in the larger project was determined using a 5-point Likert scale (1 = strongly disagree to 5 = strongly agree). These items were developed based on the standard phrasing of subjective knowledge questions [33,34,38]. Only three items regarding healthy foods applied to the present study

**Table 1:** Constructs, factors and questionnaire items.

Study construct	Factor name^a^ (number of items)	Questionnaire items
Healthy lifestyle choices	Food and sleep (items = 5)	How often do you eat fibre-rich foods, such as fruit and whole-wheat products?
		How often do you eat three full meals daily at regular times?
		How often do your meals include the basic food groups (fruits, vegetables, proteins, grains, and dairy)?
		Do you eat breakfast every day (Coffee or tea is, for the purpose of this study, not considered as a breakfast)?
		How often do you get adequate sleep (7 to 8 hours a night)?
	Exercise and relaxation (items = 5)	Do you maintain a moderate body weight for your height?
		How often do you participate in moderate exercise (such as brisk walking, low-effort cycling, mowing lawn, heavy cleaning etc.) two or three times a week?
		How often do you participate in recreational physical activities (such as hiking, swimming, bicycling or dancing)?
		How often do you get exercise during usual daily activities (such as walking during lunchtime, taking the stairs instead of the elevator; park the car away from destination and walking)?
		How often do you relax your muscles before you sleep?
		How often do you have daily relaxation time?
	Dedicated efforts (items = 3)	How often do you observe your body for abnormal changes or danger signs of possible diseases?
		How often do you seek information about your health from health professionals?
		How often do you eat preservatives in your food?
	Not smoking^b^	How often do you smoke cigarettes or pipe?
	Limit alcohol^b^	How often do you consume more than 7 alcoholic drinks per week?
Subjective knowledge of healthy food	Subjective knowledge of healthy food	Compared to an average person, I know a lot about healthy food.
		I know a lot about how to evaluate the quality of healthy food.
		People who know me, consider me as an expert in the field of healthy food.
Objective knowleddge of healthy food	Weight and cholesterol (items = 6)	Some types of dietary fibre (such as oats) lower blood cholesterol
		Most of the cholesterol in an egg is in the yellow part of the egg.
		Fish, chicken without skin, and lean meat may help to prevent obesity-related diseases.
		Polyunsaturated fats are healthier for the heart than saturated fats
		One should drink 6–8 glasses of clean water per day to maintain a healthy lifestyle.
		HDL refers to ‘good’ cholesterol, and LDL refers to ‘bad’ cholesterol
	Specific circumstances (items = 5)	There is no need to be physically active, if a person eats a healthy diet.
		The type of food a pregnant woman eats during pregnancy has no effect on the health of her unborn baby.
		The type of food a woman eats during breastfeeding has no effect on the health of her unborn baby.
		Eating breakfast is not essential, as long as one eats a balanced lunch.
		Eating a lot of salt may lower one’s blood pressure.
	Food myths and controversy (items = 6)	Even eating small amounts of starches can cause weight gain.
		Eating at fast food restaurants less than three times per week is acceptable to maintain a healthy lifestyle.
		Replacing sugar with sugar replacements, e.g. Canderall, is a better health choice.
		Trans-fats (fats found in processed food) are healthier than saturated fats (animal fats).
		It is impossible to get all of the vitamins and minerals you need from food, therefore one needs to take vitamin and mineral supplementation.
		It is acceptable to only eat two meals per day, if a person is not home during lunchtime.
		I know a lot about how to evaluate the quality of healthy food.
		People who know me, consider me as an expert in the field of healthy food.

^a^based on factor analyses.

^b^unique individual items.

The objective knowledge test regarding healthy foods was based on multiple-choice items posed to participants; they had to identify factual statements about various health-related products and issues, which were marked as correct or incorrect (1/0). This article, however, reports only on 17 items concerning healthy foods/nutrition. Considerable debate exists about what healthy food entails. Still, for this study, we referred to products included in the South African Food-based Dietary Guidelines [45] to compile the objective knowledge questions.

In our study, healthy lifestyle choices are regarded as outcome variables, while subjective knowledge, objective knowledge and demographic variables are predictors.

### Questionnaire validity and reliability

Eight experts from various disciplines within the health and related sciences were included in the expert panel (i.e., Consumer Science; Biokinetics and Wellness; Nutrition; Medical Doctor; Statistics) to establish the face and content validity of the questionnaire instruments. In addition, we held cognitive interviews to establish the questionnaire’s content, clarity, and readability [46]. We conducted exploratory factor analysis (EFA) for the adapted healthy lifestyle choice scale, subjective knowledge items, and our newly developed objective knowledge test. Principal axis factoring with Oblimin rotation was employed as the extraction method. The percentage variation explained by the EFA and Kaiser-Meyer Olkin (KMO) values was utilized to indicate construct validity and the suitability of factors. We confirmed the factor structure of healthy lifestyle choices with confirmatory factor analysis (CFA). However, due to the excellent factor structures with EFA that theoretically made sense for the new, not standardized subjective knowledge items and objective knowledge test, it was unnecessary to conduct CFA. For CFA, the root mean square error of approximation (RMSEA) with a 90% confidence interval (CI), chi-square statistic divided by degrees of freedom (χ^2^/df) and comparative fit index (CFI) served as statistical fit indices from three diverse classes [47]. The standardised regression weights (β-values) indicated the relationships of items within factors. The internal reliability of the factors was shown with Cronbach alpha values and mean inter-item correlations. The results of construct validity and internal reliability of scales are reported in the “Results from factor analyses” section.

### Data analysis

Descriptive statistics were conducted for all data, while for objective knowledge, we calculated the number of correct answers to determine the knowledge level of the participants. We calculated mean factor scores for all extracted factors from factor analyses. Missing data in measurement scales were replaced by mean-replacement. In addition, we calculated frequencies and percentages of participants adhering to the different healthy lifestyle choices from low, medium, to great extents, where low < 2.5, 2.5 < medium > 3.5, and ≥ 3.5 high. Spearman’s rank-order correlations were calculated among different factors followed by SEM to determine the associations between subjective knowledge, objective knowledge, and lifestyle choices. Due to the non-probability sample, *p*-values were not applicable, and we reported effect sizes, indicating practical significance, instead. Correlations with *r* = 0.3 (medium effect size) and *r* = 0.5 (large effect size), indicating tendencies and practical significant correlations, respectively, were reported. Demographic differences were determined with cross-tabulations, using Cramer’s V as effect sizes. Again, we only reported medium (0.3) and large (0.5) effect sizes. Spearman’s rank-order correlations were calculated for income lifestyle habits and knowledge categories. For SEM, we applied the same statistical fit indices as for CFA. IBM SPSS Statistics version 25 Release 20.0 was used for all statistical analyses except for CFA and SEM conducted with SPSS AMOS version 25.

No generalization to a larger population was made. Instead, results were interpreted only for the sample.

### Ethics

We obtained ethical clearance for this research from the Health Research Ethics Committee of the North-West University (NWU-00202-14-A1). Employees interested in participating in the study received an electronic informed consent form to provide written informed consent. They had to indicate their agreement to voluntarily participate on the electronic informed consent form before being allowed to proceed to the questionnaire.

## Results

In this paper, we discuss only the four sections of the questionnaire applicable to the present study: demographics, subjective knowledge, objective knowledge, and healthy lifestyle choices.

### Demographics

Almost half of our participants were 34 and younger, while those aged 35–44 and 45–54 were also well represented (52.4%); there were no participants older than 55 (Table 2). The majority were female, and only a third were male. Most participants were well educated, at a level higher than grade 12, and less than a quarter had completed only school level. Participants fell into three language categories: English was the most prominent home language (45.5%), followed by Afrikaans (32.0%), and African languages were in the minority (22.4%). The income distribution was predominantly dispersed among the middle income, with less than 3% of participants in the low (less than R144 000) income and approximately 10% in upper-middle to affluent (more than R600 001) income groups. the remainder of our participants belonged to a middle-income group [48]. Nearly 50% of the participants worked full 8-hour days, while an additional 45.5% had extended working hours of 9–12 hours per day. More than half of the participants also spent 2–7 hours per day working at home, including corporate and household work.

**Table 2:** Demographics of the sample (*n* = 157).

Demographic	Frequency	Percentage (Valid)
**Age (*n* = 149)**		
18–34 years	71	47.7
35–44 years	39	26.2
45–54 years	39	26.2
55 years and older	0	0.0
**Gender (*n* = 149)**		
Male	49	32.9
Female	100	67.1
**Education (*n* = 143)**		
Less than Grade 12	3	2.1
Grade 12 (school completed)	34	23.8
Diploma requiring less than three years of study	33	23.1
Degree/ Diploma requiring 3–4 years of study	52	36.4
Postgraduate	21	14.7
**Home language (*n* = 143)**		
Afrikaans	46	32.0
English	65	45.5
African languages	32	22.4
**Participant annual income bracket (ZAR)**^**a**^ **(*n* = 137)**		
Less than 72 000	1	0.7
72 001 to 144 000	3	2.2
144 001 to 252 000	49	35.8
252 000 to 420 000	54	39.4
420 001 to 600 000	16	11.7
600 001 to 960 000	8	5.8
960 001 and above	6	4.4
**Hours at work (*n* = 134)**		
Half a day	7	5.2
Full day	66	49.3
9 to 12 hours	61	45.5
**Hours spend on work at home (*n* = 136)**		
None	66	48.5
Less than 2 hours	41	30.1
2 to 4 hours	14	10.3
5 to 7 hours	15	11.0

^a^ZAR: 1 ZAR = approximately 0.054 US$.

### Results from factor analyses

**Fig 1:**
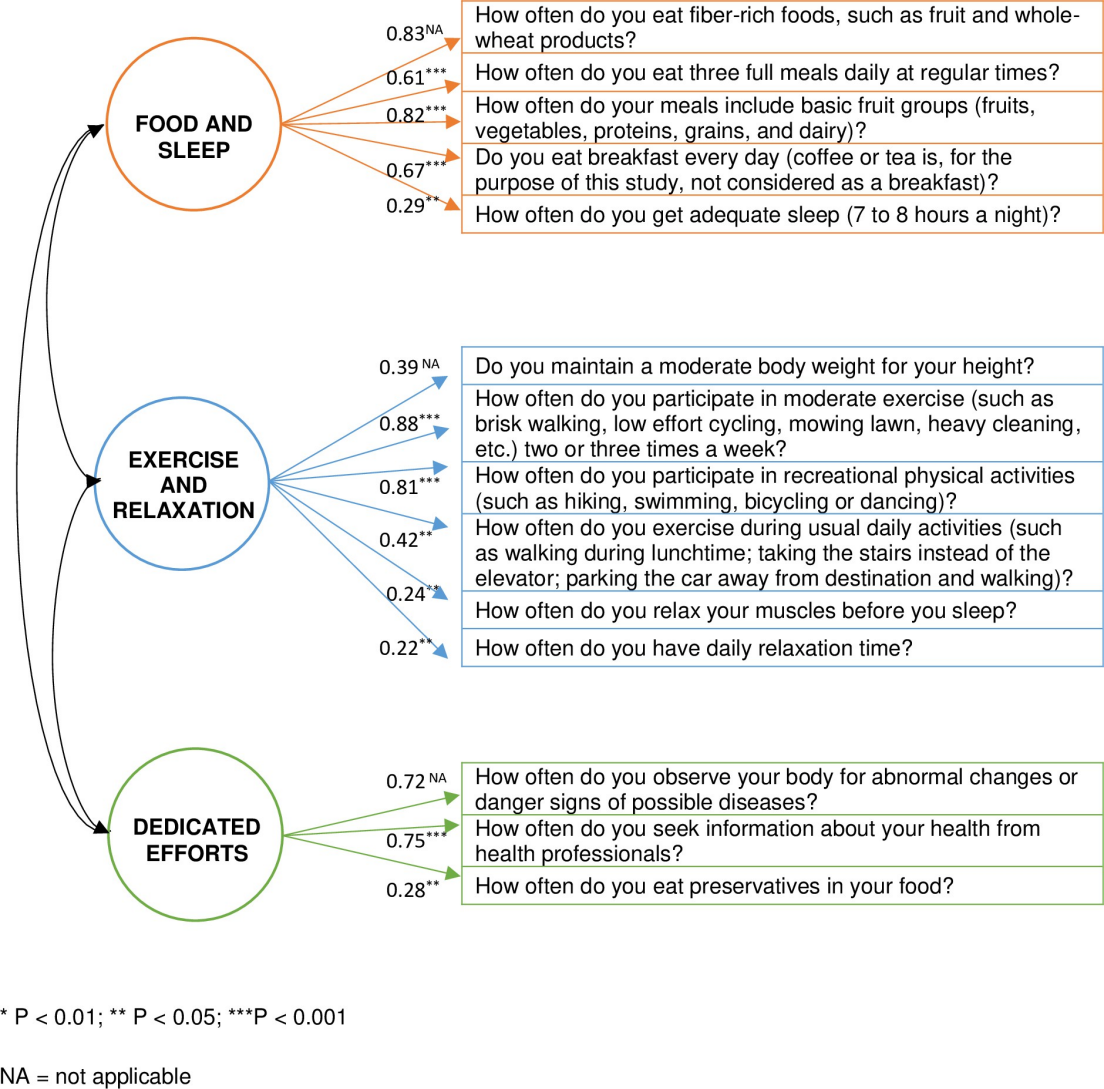
Confirmatory factor analysis for healthy lifestyle habits.

Exploratory factor analysis extracted three reliable factors for lifestyle choices, namely, “food and sleep” (Cronbach α = 0.787), “dedicated efforts” (α = 0.603), and “exercise and relaxation” (α = 0.671), which aligned well with theory and had mean interitem correlations of 0.423, 0.321 and 0.251, respectively (Table 1). These factors were reliable based on their Cronbach alpha values, more than the minimum value of 0.6 [49] and inter-item correlations ideally being between 0.15 and 0.55 [50]. The three-factor EFA explained an acceptable 49.8% of the variance and had a KMO of 0.70. For the food and sleep factor, all items entailed healthy food-related habits, except for the sleep item, but which strongly loaded unto this factor probably since all related to familiar food and sleep-related practices; hence the name “food and sleep”. Dedicated efforts as a factor entailed additional effort that consumers might expend to pursue health, such as health-professional consultations, self-examination for disease, and avoidance of food preservatives. Finally, exercise and relaxation entailed recreational activities, exercise, relaxation, and weight control. Additionally, two unique items, “Not smoking” and “Limit alcohol”, did not fit the factor structures but were important as healthy lifestyle choices and retained as individual items. For the CFA, most standardised regression weights (β = 0.390–0.884) were above 0.3, except for a few ranging between 0.217 and 0.287. However, all loadings of items onto the factors were significant at *p* < 0.001 or, in a few cases, *p* < 0.05. Two of the three statistical indices were acceptable, with RMSEA = 0.07 [90% CI = 0.054–0.093] lower than 0.1 [51] and χ^2^/df = 1.84 between 1 and 5, while CFI = 0.87 was close to 0.9 instead of larger than 0.9 [52]. Two of the three indices were good, and the final one was close to acceptable; thus, we could confirm the model’s goodness of fit. Fig 1 depicts the CFA model.

The EFA on the subjective knowledge items yielded one factor with KMO = 0.695 and explained a notably high 79.74% of the variance. Thus, construct validity was confirmed, and the single factor named “subjective knowledge of healthy foods” had good internal reliability based on Cronbach α = 0.868. However, the mean inter-item correlation (0.695) was slightly higher than the recommended 0.55 [50], attributed to the similarity of the three items compiling this factor.

Exploratory factor analysis for objective knowledge of healthy food/nutrition extracted three factors explaining an acceptable 49.24% of the variance with a KMO value of 0.752, thereby confirming construct validity. The first factor, “weight and cholesterol” (α = 0.868; mean inter-item correlation = 0.515), pertained to cholesterol-containing and -lowering foods, fat-containing foods and obesity. The second factor, “specific circumstances” (α = 0.686; mean inter-item correlation = 0.329), contained questions regarding healthy food and behaviours related to circumstances, including physical activity, pregnancy, and blood pressure. Finally, “food myths and controversy” (α = 0.614; mean inter-item correlation = 0.218) related to myths about food consumption that consumers may believe. These included, for example, weight gain through the intake of even small amounts of starch, limiting fast food intake to three times a week, replacing animal fats with trans fats in processed foods, and skipping meals. It also included controversial issues such as sugar replacements and nutritional supplements. These factors were reliable based on their Cronbach alpha values and inter-item correlations.

### Healthy lifestyle choices

Table 3 presents the participants’ mean factor score values for the five identified healthy lifestyle choices. On average, participants ‘often’ adhered to non-smoking and limited alcohol drinking (1 = never to 5 = often), while lifestyle choices concerning food and sleep, dedicated efforts and exercise and relaxation were only ‘seldom’ followed. In terms of low, medium, and high frequencies of adherence to lifestyle choices, almost 70% habitually avoided smoking and limiting drinking alcohol. Although less than half (44.4%) of the participants showed high commitment to healthy food and sleep choices, they adhered better to this habit than habits of dedicated efforts and exercise and relaxation. Almost half (48.1%) were in the medium range for dedicated efforts. Exercise and relaxation were adhered to the least (50.4%), with half of the participants in the low range.

**Table 3:** Descriptives for healthy lifestyle habits.

Healthy lifestyle habits^a^	Mean factor scores (SD^c^)	95% CI^d^ lower	95% CI^d^ upper	Low^e^		Medium^e^		High^e^	
				*n*	%	*n*	%	*n*	%
Not smoking^b^ (*n* = 131)	4.01 (1.43)	3.76	4.26	31	23.7	9	6.9	91	69.5
Limit alcohol^b^ (*n* = 131)	3.95 (1.20)	3.74	4.16	19	14.5	22	16.8	90	68.7
Food and sleep *(n* = 133)	3.27 (0.86)	3.12	3.42	27	20.3	47	35.3	59	44.4
Dedicated efforts *(n* = 131)	2.99 (0.77)	2.86	3.13	35	26.7	63	48.1	33	25.2
Exercise and relaxation (*n* = 131)	2.60 (0.67)	2.49	2.72	66	50.4	47	35.9	18	13.7

^a^Healthy lifestyle habit scale: 1 = never, 2 = seldom, 3 = sometimes, 4 = often, 5 = always.

^b^Unique individual items with mean values.

^c^Standard deviation.

^d^Confidence interval.

^e^Low values ≤ 2.5; medium = 2.5 to ≤ 3.5, high ≥ 3.5.

Cross tabulations revealed differing tendencies (i.e., medium effect sizes) among age groups for levels of exercise and relaxation participation (Cramer’s V = 0.228) and for alcohol consumption (Cramer’s V = 0.249). More participants who were 45–54 years (30.6%) tended to engage in high levels of exercise and relaxation habits than younger participants (18–34 years = 8.1%; 35–44 years = 6.1%). Furthermore, although most participants in all age groups largely limited alcohol consumption, the highest proportion was among the group aged 35–44 years (90.9%), that is, higher than those who were younger or older (18−34 years = 56.5%; 45−54 years = 69.4%). The tendency to limit alcohol consumption also differed by gender (Cramer’s V = 0.259), with 75.6% of female participants having a higher level of limiting alcohol usage, in contrast to 55.6% among males.

Educational level differences were also echoed regarding exercise and relaxation (Cramer’s V = 0.22). More participants with postgraduate qualifications (63.2%) tended to exercise and relax at a medium level. In comparison, those with lower qualification levels also tended to engage in exercise and relaxation at a lower level (grade 12 and lower = 51.5%; diploma [< 3 years] = 66.7%; degree/diploma [3–4 years] = 53.1%).

Participants’ income correlated with medium effect sizes with healthy lifestyle choices regarding food and sleep, dedicated efforts and exercise and relation, respectively. The higher the income level, the more participants engaged in these lifestyle choices (Table 4).

**Table 4:** Spearman’s correlations (*r*) of income, healthy lifestyle choices and subjective and objective knowledge about healthy foods.

	HL: Food & sleep	HL: dedicated efforts	HL: Exercise & relaxation	HL: Not smoke	OK: Weight & cholesterol	OK: Specific circum-stances	OK: Food myths & contro-versy
**Income**	0.271	0.246	0.277	-	-	-	-
**HL** ^**a**^ **: Food & sleep**	1	0.212	0.464	-	-	-	-
**HL: Dedicated efforts**	0.212	1	0.262	-	-	-	-
**HL: Limit alcohol**	-	-	-	0.275	-	-	-
**SK** ^**b**^ **: Healthy foods**	0.396	0.382	0.373	0.351	-	-	-
**OK** ^**c**^ **: Weight & cholesterol**	-	-	-	-	1	-0.307	-0.543
**OK: Specific circumstances**	-	-	-	-	-	1	0.352
**OK: Food myths & controversy**	-	-	-	-	-	0.352	1

^**a**^HL: Healthy lifestyle choices.

^**b**^SK: Subjective knowledge.

^**c**^OK: Objective knowledge.

*r* = 0.3 –medium effect size; *r* = 0.5 –large effect size; small effect sizes not reported.

### Subjective knowledge about healthy foods

The mean factor score for subjective knowledge (*n* = 96) about healthy foods based on the three items was 3.59 ± 0.83 (95%CI [3.42, 3.76]). This showed that the participants, on average, viewed themselves as knowledgeable about healthy foods (1 = strongly disagree to 5 = strongly agree). Furthermore, 58.3% showed a high level of subjective knowledge, followed by 28.1% with a medium and 13.5% with a low level of subjective knowledge.

Cramer’s V values revealed differences with medium effect sizes in subjective knowledge by gender (Cramer’s V = 0.29) and qualifications (Cramer’s V = 0.29). More females (60.3%) than males (54.5%) had high subjective knowledge levels. Concerning education, more participants from the two highest qualification categories (degree or diploma 3–4 years = 63.9%; a postgraduate degree = 80.0%) had higher levels of subjective knowledge than those with lower qualifications (Grade 12 and lower = 52.0%; diploma less than three years = 40.0%).

### Objective knowledge about healthy foods

Our results showed that participants, on average, had excellent objective knowledge in all the categories, except the one relating to weight and cholesterol, with less than 40% of correct responses (Table 5). The analysis revealed that over 90% of participants were highly knowledgeable regarding healthy foods in context-specific circumstances (e.g., physical activity, pregnancy, and blood pressure), food myths, and controversial issues. However, over half fell into the low objective knowledge category regarding weight and cholesterol. We found no demographic differences among objective knowledge levels.

**Table 5:** Descriptives for objective knowledge of healthy foods.

Objective knowledge	Mean % correct^a^ (SD^b^)	95% CI^c^ lower	95% CI^c^ upper	Low^d^		Medium^d^		High^d^	
				*n*	%	*n*	%	*n*	%
Specific circumstances (*n* = 157)	95.92 (13.15)	93.82	98.02	3	1.9	2	1.3	152	96.8
Food myths and controversy (*n* = 157)	88.43 (18.17)	85.53	91.33	4	2.5	8	5.1	145	92.4
Weight and cholesterol (*n* = 157)	36.73 (36.43)	30.91	42.55	80	51.0	19	12.1	58	36.9

^a^Mean % correct responses to questions within the factors; Objective knowledge measured as correct = 0 and incorrect = 1.

^b^Standard deviation.

^c^Confidence interval.

^d^Low values < 40% correct responses; medium = 40–60%, high > 60%.

### Associations between healthy lifestyle choices, subjective knowledge, and objective knowledge

Table 4 depicts the correlations between the different constructs. The three healthy lifestyle choices relating to ‘food and sleep’, ‘dedicated efforts’, and ‘exercise and relaxation’ correlated with medium to large effect sizes (*r =* 0.212 to 0.464). The ‘not smoking’ choice correlated with ‘limit alcohol’. Subjective knowledge about healthy foods did not correlate with any objective knowledge category. Only the different factors regarding objective knowledge about healthy foods correlated, with a positive correlation between ‘specific circumstances’ and ‘food myths and controversy’. Conversely, objective knowledge regarding ‘weight and cholesterol’ showed negative correlations with the other two knowledge categories (Table 4).

Finally, and most important for this study, we focused on correlations concerning subjective and objective knowledge about healthy foods with healthy lifestyle choices. Participants’ subjective knowledge correlated practically significantly with all healthy lifestyle choices, except ‘limit alcohol’. However, we found no correlations between objective knowledge and healthy lifestyle choices (Table 4).

**Fig 2:**
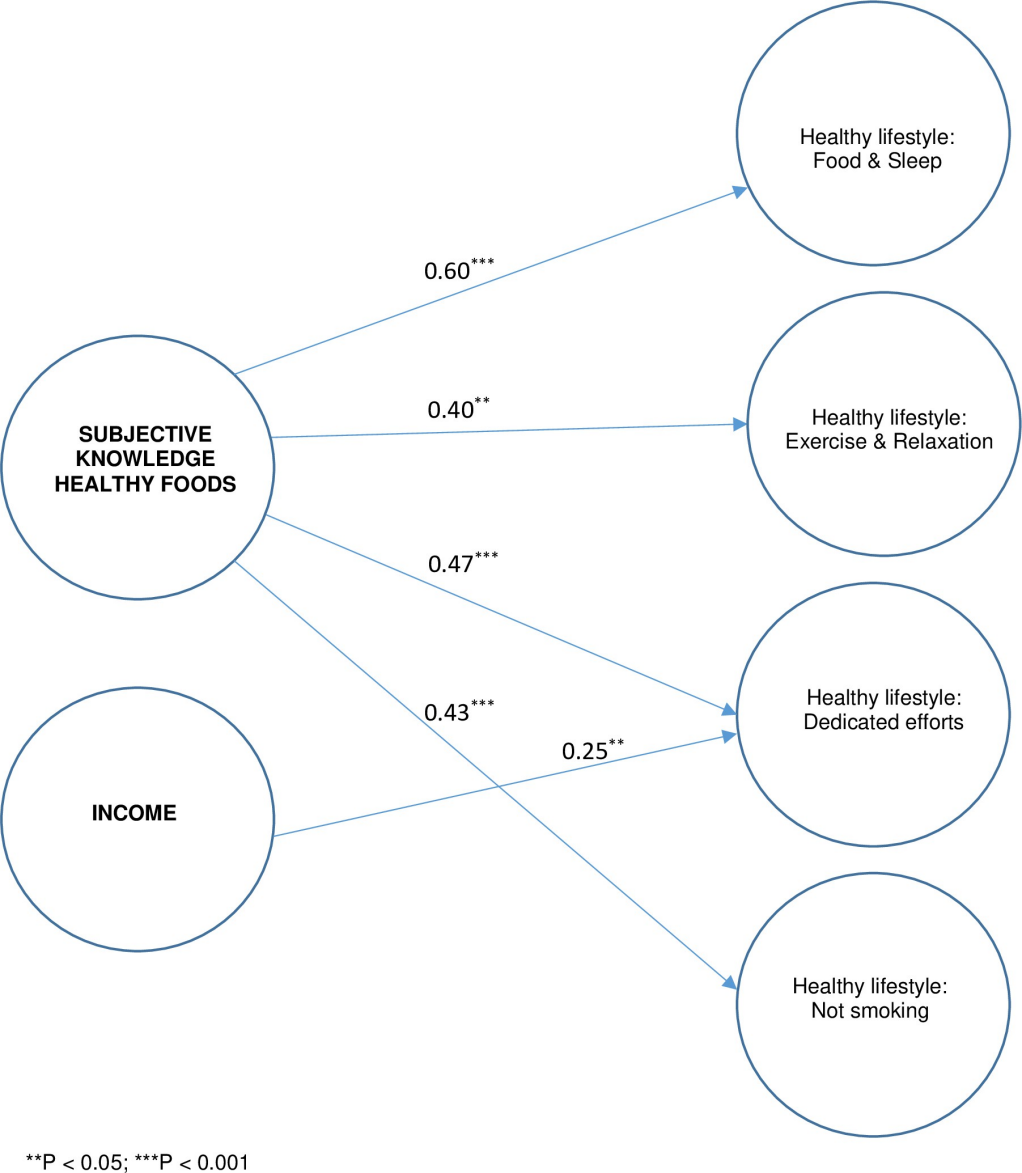
Final structural model explaining the association between subjective knowledge of healthy foods and healthy lifestyle habits.

Based on the above correlations, our first structural equation model to determine the relationship between healthy lifestyle choices and knowledge excluded objective knowledge since objective knowledge categories failed to correlate with healthy lifestyle choices. Due to its correlation with healthy lifestyle choices, income was also included in the model. The proposed model tested the contribution of subjective knowledge about healthy foods, as well as income, to all remaining healthy lifestyle choices. The model fit was acceptable according to two of the three goodness of fit indices with good values for RMSEA = 0.071 [0.058–0.084] and χ^2^ /df = 1.795, with CFI = 0.821, slightly low. However, the standardized regression weights between subjective knowledge and limiting alcohol were not statistically significant (β = 0.047; *p* = 0.635). Therefore, for the final model, limiting alcohol was omitted as a healthy lifestyle choice. Fig 2 depicts the structural part of the final model. All standardized regression weights (0.40–0.60) of subjective knowledge loading onto the different healthy lifestyle factors were significant at *p* < 0.001 or *p* < 0.05, with income significantly (*p* = 0.012) loading only onto dedicated effort choices. As with the proposed model, two of the three statistical indices were acceptable with RMSEA = 0.065 [0.051–0.080], χ^2^ /df = 1.666 and CFI = 0.861; therefore, we could confirm the goodness of the model’s fit. According to the standardized regression weights in Fig 2, subjective knowledge made the most considerable unique contribution to lifestyle choices concerning food and sleep, followed by dedicated efforts. The contribution of subjective knowledge to ‘not smoking’ and ‘exercise and relaxation’ was the smallest and almost equal, with income making the smallest contribution to the final model.

## Discussion

Our study sample comprised mainly young and middle-aged, urban, well-educated participants with regular to relatively long working hours in a corporate setting. The high proportion (almost three-quarters) of 18–44-year-old participants and the low proportion of earners in the lowest and highest income brackets point to a group of professionals building their careers with potentially busy, time-restricted lives and, consequently, the potential for less-than-optimal healthy lifestyle choices. Their employment status led to an underrepresentation of low-income consumers.

Our participants’ healthy-lifestyle-habit profile demonstrated suboptimal adherence to crucial healthy lifestyle choices other than avoidance of smoking and limiting alcohol and reiterated the growing risk for cardiovascular diseases. As expected, half of the participants fell in the low range of participation in exercise and relaxation since these activities may place additional demands on their time and expenses. This result echoes research among African countries’ executives, showing severely deficient physical activity levels [53]). Previous studies warn against the adverse effects of insufficient physical activity among corporate staff, including productivity losses, inflated healthcare costs, and employee morbidity and mortality [43,53]. Additionally, physical activity has been shown as an effective strategy for promoting well-being during the COVID-19 lockdown [54].

The greater number of participants with high adherence to exercise and relaxation healthy lifestyle choices among the older group aged 45–54 years, in contrast with lower commitment among the younger age groups, was surprising. However, it is possible that having reached a later stage in their careers, family life and social responsibilities; they now had more time or freedom for these activities. Moreover, being older, they may have experienced declining physical endurance or have been warned by medical practitioners about the importance of exercise and relaxation choices in maintaining health; their higher restriction of alcohol may also reflect such recognition. Studies have shown that even moderate exercise engagement and physical activity have improved health and productivity among older employees [43] and increased satisfaction-with-life levels [55]. Thus, amid the prospects of declining age-associated cardiovascular health in South Africans [56], the tendencies shown in our study among older participants are encouraging and could become even more so if such choices somehow spill over to other age categories. Although our analysis revealed tendencies (i.e., medium effect sizes) of differences between qualification levels concerning lifestyle choices about exercise and relaxation, none of the age groups excelled in these choices at a high level.

Our study’s ‘dedicated efforts’ lifestyle choices covered additional conscious efforts made by participants to maintain health. Relevant actions, namely, limiting the intake of food preservatives, seeking health information from health professionals, and observing one’s body for abnormal signs of possible disease, presented a further healthy-lifestyle challenge for our participants. Again, less than half of them adhered even to a medium extent to such choices, possibly because these demand extra time and effort. Suki [57] showed somewhat different results among Malaysian students practising ecological behaviour. They largely adhered to choices, including avoiding additives and precooked food and limiting salt and red meat intake. These studies’ different contexts of corporate workers versus students can explain these contradictions but offer opportunities for expanding ecological behaviour research to broader contexts.

Most of our participants managed to refrain from smoking and limiting alcohol to a high degree. However, more females limited their alcohol use to a high level than males. Additionally, more participants adhered to a high level of healthy choices regarding food and sleep (compared to dedicated efforts, exercise, and relaxation), despite the time and devotion demands of healthy food and sleep choices. However, they still made up less than half of the participants. We can draw some parallels from former research with our participants. Casini et al. [2] identified poor lifestyle choices favouring animal protein and convenience food consumption among Italian consumers from middle to high-income and education levels.

In terms of reaching out more broadly, the literature offers insights into public awareness campaigns and interventions that have been successful in food consumption and smoking [58–60]. Therefore, public awareness initiatives could be valuable by following similar approaches, targeting neglected healthy lifestyle choices, including exercise, relaxation, and dedicated efforts. However, studies have cautioned about the difficulty of persuading people to exercise and stick to it [43] and the challenges experienced by campaigns persistently changing unhealthy choices [60] and is primarily dependent on personal willpower to make such changes [61]. Therefore, the avenues of approaching public health initiatives towards healthy lifestyle choices should be carefully selected and offer participant support.

On average, our participants had high subjective knowledge about healthy foods, with most perceiving themselves as highly knowledgeable. Female participants’ tendency towards higher subjective knowledge than their male counterparts supports other research showing females as more health-conscious than males [62]. Women are also often the household food decision-makers [63], which could help to explain their higher confidence levels in their knowledge about healthy foods. Earlier studies of subjective knowledge revealed gender and educational level differences for subjective knowledge on other topics, with differences in subjective knowledge about the human papillomavirus [32]. Furthermore, our finding that subjective knowledge tended to increase with higher qualification levels supports the literature that reports similar results on genetically modified foods [35] and nutrition label information [39]. However, they did not report gender differences.

Unexpectedly, participants revealed excellent objective knowledge about healthy foods and nutrition relating to specific circumstances, myths, and controversies. Their knowledge level regarding particular circumstances, including physical activity, pregnancy, breastfeeding and hypertension, pointed to a genuine interest in eating habits and nutrition in these contexts. A similar trend emerged in their objective knowledge about key food myths and controversial food-related issues; our participants were noticeably able to sieve information and hold on to scientifically proven evidence regarding typical food myths doing the rounds and more complex issues such as sugar replacements and nutritional supplements. Lewandowsky et al.’s [64] review addressing misinformation communicated to consumers revealed that cognitive factors related to intelligence, memory, and capacity to tolerate ambiguity might mediate the effect of misinformation on recipients. In this light, our participants’ high objective knowledge about food myths and controversial issues across all demographics may relate to their relatively high overall education levels.

Almost half of our participants fell in the low objective knowledge category regarding the effects on health and obesity of cholesterol-containing/lowering foods and fats. This finding echoes contradictory messages such as those relating to the consumption of eggs and the butter–margarine debate. We regard this objective knowledge gap as the most severe concern arising from our study, given escalating obesity across lower to high-income countries [12] and the recognised relationship with and dietary contribution of saturated fat to hypercholesterolaemia and cardiovascular disease [45]. Participants’ limited objective knowledge in these areas could mirror confusion and misconceptions about dietary fat intake due to conflicting media and scientific messages. In their literature review, Liu et al. [65] evaluated consumer confusion amid scientific evidence relating to sources of dietary fat and types of fat in the context of cardiovascular risk reduction. Our findings support the views of these authors that conflicting messages and misconceptions about food and health can harm consumers’ health. In the case of our sample of relatively young adult participants, insufficient objective knowledge about healthy foods and their effects on weight and cholesterol is concerning.

Our participants’ subjective and objective knowledge profiles revealed that what they thought they knew about healthy foods did not always reflect objective scientific facts. However, this lack of correlation between objective and subjective knowledge was not unexpected since the literature has often pointed to inconsistencies between the two [e.g., 35,39]; discrepancies can result when individuals over- or under-estimate their actual knowledge of the facts [29,35].

In our study, subjective knowledge about healthy foods was associated with healthy lifestyle choices, but we found no such association with objective knowledge. Similarly, the literature indicates a stronger association of subjective knowledge than objective knowledge regarding various other health-related behaviours [32,37,38,40]. This correlation suggests that in interventions to reduce the risks of NCDs and address obesity, consumers with higher subjective knowledge of healthy foods are more likely to participate actively in healthy lifestyle choices.

Our research reveals the contribution of subjective knowledge to healthy lifestyle choices and the extent of this contribution to lifestyle choices. SEM showed that our participants’ subjective knowledge about healthy foods was associated with healthy lifestyle choices of food and sleep, dedicated efforts, exercise and relaxation, and not smoking. In addition, we demonstrated that subjective knowledge about healthy food contributed more pronounced to food and sleep and dedicated efforts than to the other choices. We ascribe the more significant contribution of subjective knowledge to their relevance to the relevance of these lifestyle choices to food-related aspects of health. Besides the fact that food and sleep habits are directly connected with eating and drinking, dedicated efforts include limiting the intake of food preservatives and obtaining information from health professionals, amongst whom nutritionists and dieticians feature. These findings add to earlier studies that revealed the influence of subjective knowledge on consumer behaviours [e.g., 32,37,39,40]. The relationship we found between subjective knowledge and the other lifestyle choices of avoiding smoking and engaging in exercise and relaxation was unexpected. However, it was promising for future research into factors associated with healthy lifestyle choices and for designing consumer education interventions.

Participants’ income was associated with healthy lifestyle choices regarding food and sleep, exercise and relaxation and dedicated efforts. However, income only made a statistically unique contribution to lifestyle choices requiring dedicated efforts according to our structural model. Such dedicated efforts may require a premium cost, such as visiting health professionals, or only purchasing food without preservatives. Although our findings highlight the relationships between subjective knowledge about healthy foods with participants’ specific healthy lifestyle behaviours, and income made a lesser contribution to one lifestyle choice category, it remains essential to consider consumer decisions in a broader context. Factors in the consumer’s external environment, such as the price and availability of healthy food, over-availability of unhealthy, ultra-processed products, and pervasive marketing techniques used by the food and beverage industry, influence healthy food and lifestyle choices. Although outside the scope of our study, they may have a greater impact on lifestyle choices than subjective knowledge of healthy behaviours. The contribution of income to participants’ dedicated effort choices illustrates the importance of such an external factor. However, our findings focusing on the two knowledge categories allow for valuable health promotion recommendations.

Though not shown to be associated with specific healthy lifestyle choices, the low level of objective knowledge regarding weight and cholesterol we identified in our study should be addressed. The inappropriate or excessive consumption of energy-dense or cholesterol-containing foods is a matter of public health.

## Limitations and strengths

The research reported here provides opportunities for further studies in consumer behaviour, public health, and consumer education in addressing NCD threats. The study findings are confined to a specific corporate setting in urban South Africa; our sample size was relatively small, with a limited representation of lower-qualified, low-income employees and older and high-income executives. Furthermore, we did not attempt to establish causality between knowledge about healthy foods and healthy lifestyle choices, and our sample needed to be randomized for the findings to be generalizable. Access to an existing wellness programme may have benefitted our participants’ knowledge and lifestyle choices. It may have made them more amenable to participating than their unhealthy and/or unknowledgeable counterparts, given our use of an availability sample. However, despite this programme, they showed limited adherence to some lifestyle choices and severe knowledge deficiencies. Therefore, this study offers a valuable baseline for research into objective knowledge, subjective knowledge and healthy lifestyle choices.

## Translation into practice

This research can benefit researchers, practitioners, and consumers in revealing objective knowledge and lifestyle challenges consumers experience and opportunities to address these. Despite the findings of this study not being generalizable, they can support current intervention programmes. The current PC 101 guideline and ICDM model follow generic processes for health promotion. Still, this study shows that the demographic uniqueness of a consumer must be considered in health education to improve its effectiveness, even more so in South Africa, with an unequal and diverse society. We showed that healthy lifestyle choices regarding exercise and relaxation across all educational levels might require intervention. Our findings suggest that campaigns encouraging exercise and relaxation could concentrate on reaching younger age categories. In contrast, dedicated effort in lifestyle choices could warrant initiatives that extend more broadly across age categories.

Furthermore, this study revealed that consumers’ subjective knowledge about healthy foods, though not always reflective of accurate, objective knowledge, may need focused attention when approaching healthy lifestyle interventions. Despite our participants’ generally high levels of subjective knowledge about healthy foods, our results suggest that interventions to boost subjective knowledge could be directed predominantly towards males and demographics with lower qualifications. Given that subjective knowledge is associated with self-confidence in one’s knowledge [29], such interventions could be designed to include healthy-food-related decision tasks and building experience and self-belief among males and lower-qualified participants regarding their knowledge of healthy food choices. Such tasks can expose them to the kind of awareness that women naturally gain in their roles as frequent household food purchasers.

In practice, our study suggests that consumer education or health promotion strategies should focus on increasing subjective knowledge about healthy food―the amount of knowledge consumers think or perceive themselves to have about healthy food. However, these strategies should also incorporate and transfer scientific facts about fat, energy and cholesterol intake and their link with health concerns to align subjective and objective knowledge. Thus, interventions need to pay less attention to areas where objective knowledge is high and more where it is low. The media has a significant part to play in communicating health-related information to consumers. However, to combat misinformation or half-truths, interventions must focus on conveying essential science-based information—for example, about cholesterol-containing/preventing foods and fats and their effects on health and weight management. Lewandowsky et al. [64] warn that consumers struggle to change what they have learned based on misinformation, even after such information has been retracted, making it challenging to reverse or override existing misconceptions. Misinformation, particularly regarding healthy fat intake, cholesterol, and obesity, deserves corrective actions and emphasis from the media, health practitioners, and consumer educators; otherwise, misinformation can undermine efforts combatting NCDs. For consumers, such as those in our study, interventions and education can be embedded in company wellness programmes. These programmes could play a fundamental role in educating consumers across all demographics by focusing on tasks distinguishing fact from fiction and working with complex purchasing-decision scenarios. Schouw and Mash [66] revealed promising improvements in NCD-risk factors in a South African workplace when introducing a multi-component wellness intervention programme, with a significant reduction in alcohol consumption, improved fruit and vegetable intake, and physical activity. We thus recommend that objective knowledge be covered in such programmes, especially those on healthy food, related directly to the chief NCDs of concern for corporate employees’ health vulnerabilities.

Health-related practitioners involved in corporate wellness programmes and other role players in consumer education could apply our subjective and objective knowledge findings to existing or new programmes. Equipping consumers with the necessary objective and subjective knowledge regarding healthy foods, they could, in due course, pursue more beneficial lifestyle choices regarding food and even other decisions.

## Future research

The present study did not investigate the reasons for participants’ adherence or neglect of particular lifestyle choices, which could offer health scientists valuable avenues for future investigation. Further research should focus on the age group of 45−54, which could lead to a greater understanding of these consumers’ motivations to engage more in exercise and relaxation while offering possible approaches to encourage younger age groups to improve their adherence to these healthy lifestyle choices.

However, to the best of our knowledge, our study is the first to examine the practice of healthy lifestyle choices from the perspective of consumers’ subjective and objective knowledge about healthy foods using SEM. We implemented a more comprehensive adapted healthy lifestyle habit instrument. This scale and our developed objective knowledge instrument offer opportunities for implementation in future research. Our structural model was the first to highlight the contribution of subjective knowledge about healthy foods to specific lifestyle choices. Although these scales and this model need further testing, they can serve as a guideline tool to build on with the future investigation of correlations and comparisons when repeated with different cohorts.

Future endeavours can explore different contexts and more extensive, representative samples, as findings relating to less urban, lower-income or lower-literate consumers may differ from ours. Further investigation into objective knowledge components among larger samples and a broader range of demographics could inform targeted interventions that suit disparate contexts and needs. In addition, it would be valuable to incorporate consumer self-confidence as a construct in the relationships between subjective knowledge of food and healthy lifestyle choices in future research.

## Conclusion

Consumer knowledge of healthy foods, healthy food choices and healthy lifestyle behaviours play an essential role in combatting NCDs and supporting public health. Our findings revealed challenges among participants in making lifestyle choices concerning food and sleep, dedicated efforts and exercise and relaxation, and needing more knowledge about healthy foods relating to fat and cholesterol-related aspects. We also showed that objective knowledge of healthy foods alone does not support healthy lifestyle choices. Instead, we showed consumers’ subjective knowledge about healthy foods, though not always reflective of accurate, objective knowledge, deserves focused attention when approaching healthy lifestyle interventions.

## Supporting information

S1 Appendix Questionnaire. Questionnaire questions applicable.
(PDF)

S2 Appendix Data. Applicable study data.
(XLSX)
