# Supplementary material for: Consumer objective and subjective knowledge about healthy foods: An approach to promote healthy lifestyle choices in South Africa
Source: PLoS One. 2024 Jan 25;19(1):e0296504. doi: 10.1371/journal.pone.0296504 (PMC10810447; doi:10.1371/journal.pone.0296504)
Supplement: S1 Appendix — Questionnaire questions applicable. (PDF) [file pone.0296504.s001.pdf]

APPENDIX 1: Applicable questionnaire questions

Demographics

1. How old are you?
   1. 18-24 years
   2. 25-34 years
   3. 35-44 years
   4. 45-54 years
   5. 55-64 years
   6. 65-75 years
   7. Older than 75 years

2. What is your gender?
   1. Male
   2. Female

3. What is your highest qualification?
   1. Less than grade 10
   2. Grade 10
   3. Less than grade 12
   4. Grade 12
   5. Diploma requiring less than 3 years of study
   6. Degree/Diploma requiring 3 to 4 years of study
   7. Post-graduate

4. What is your first language?
   1. Afrikaans
   2. English
   3. IsiNdebele
   4. IsiXhosa
   5. IsiZulu
   6. Sesotho sa Leboa
   7. Sesotho
   8. Setswana
   9. SiSwati
   10. Tshivenda
   11. Xitsonga
   12. Other ___________________________________

5. In which salary bracket does your income fall?
   1. less than R72 000 annually
   2. R72 001 - R144 000 annually
   3. R144 001 - R252 000 annually
   4. R252 001 - R420 000 annually
   5. R420 001 - R600 000 annually)
   6. R600 001 - R960 000 annually
   7. R960 001 - R1 200 000 annually
   8. R1 200 001 - R1 800 000 annually

9. more than R1 800 000 annually

6. How many hours per day, on average, do you spend at work?
    1. Less than half a day
    2. Half a day
    3. Full day
    4. 9 to 12 hours
    5. 13 to 16 hours
    6. More than 16 hours

7. How many hours per day, on average, do you spend on work at home?
    1. None
    2. Less than 2 hours
    3. 2 to 4 hours
    4. 5 to 7 hours

Healthy lifestyle choices

8. How often do you do the following?

	1 Never	2 Seldom	3 Sometimes	4 Often	5 Always
1. How often do you eat three full meals daily at regular times?	❑	❑	❑	❑	❑
2. How often do your meals include the basic food groups (fruits, vegetables, proteins, grains, and dairy)?	❑	❑	❑	❑	❑
3. Do you eat breakfast every day (Coffee or tea is, for the purpose of this study, not considered as a breakfast)?	❑	❑	❑	❑	❑
4. How often do you eat fibre-rich foods, such as fruit and whole-wheat products?	❑	❑	❑	❑	❑
5. How often do you eat preservatives in your food?	❑	❑	❑	❑	❑
6. How often do you participate in moderate exercise (such as brisk walking, low-effort cycling, mowing lawn, heavy cleaning etc.) two or three times a week?	❑	❑	❑	❑	❑
7. How often do you participate in recreational physical activities (such as hiking, swimming, bicycling or dancing)?	❑	❑	❑	❑	❑
8. How often do you get exercise during usual daily activities (such as walking during lunchtime, taking the stairs instead of the elevator, park the car away from destination and walking)?	❑	❑	❑	❑	❑
9. How often do you get adequate sleep (7 to 8 hours a night)?	❑	❑	❑	❑	❑
10. How often do you relax your muscles before you sleep?	❑	❑	❑	❑	❑

11. How often do you have daily relaxation time?	❑	❑	❑	❑	❑
12. How often do you smoke cigarettes or pipe?	❑	❑	❑	❑	❑
13. Do you maintain a moderate body weight for your height?	❑	❑	❑	❑	❑
14. How often do you observe your body for abnormal changes or danger signs of possible diseases?	❑	❑	❑	❑	❑
15. How often do you seek information about your health from health professionals?	❑	❑	❑	❑	❑
16. How often do you consume more than 7 alcoholic drinks per week?	❑	❑	❑	❑	❑

Subjective knowledge

9. To which extent do you agree with the following statements about yourself? ...

	1 Strongly disagree	2 Disagree	3 Tend to agree	4 Agree	5 Strongly agree
1. COMPARED TO AN AVERAGE PERSON, I KNOW A LOT ABOUT healthy food	❑	❑	❑	❑	❑
2. I KNOW A LOT ABOUT HOW TO EVALUATE THE QUALITY OF healthy food	❑	❑	❑	❑	❑
3. PEOPLE WHO KNOW ME, CONSIDER ME AS AN EXPERT IN THE FIELD OF healthy food	❑	❑	❑	❑	❑

Objective knowledge

10. Which statement about lifestyle and nutrition is TRUE? SELECT ALL THE CORRECT ANSWERS.
    a. There is no need to be physically active, if a person eats a healthy diet
    b. One should drink 6 – 8 glasses of clean water per day to maintain a healthy lifestyle
    c. Even eating small amounts of starches can cause weight gain
    d. Eating breakfast is not essential, as long as one eats a balanced lunch

11. Which statement about nutrition is TRUE? SELECT ALL THE CORRECT ANSWERS.
    a. Eating at fast food restaurants less than three times per week is acceptable to maintain a healthy lifestyle
    b. Some types of dietary fibre (such as oats) lower blood cholesterol
    c. Replacing sugar with sugar replacements, e.g. Canderall, is a better health choice

12. Which statement about nutrition is TRUE? SELECT ALL THE CORRECT ANSWERS.
    a. Trans-fats (fats found in processed food) are healthier than saturated fats (animal fats)
    b. It is impossible to get all of the vitamins and minerals you need from food, therefore one needs to take vitamin and mineral supplementation
    c. Most of the cholesterol in an egg is in the yellow part of the egg

13. Which statement about nutrition is TRUE? SELECT ALL THE CORRECT ANSWERS.
    a. Fish, chicken without skin, and lean meat may help to prevent obesity-related diseases
    b. Polyunsaturated fats are healthier for the heart than saturated fats

c. The type of food a pregnant woman eats during pregnancy has no effect on the health of her unborn baby
d. The type of food a woman eats during breastfeeding has no effect on the health of her baby

14. Which statement about nutrition is TRUE? SELECT ALL THE CORRECT ANSWERS.
a. HDL refers to ‘good’ cholesterol, and LDL refers to ‘bad’ cholesterol
b. It is acceptable to only eat two meals per day, if a person is not home during lunchtime
c. Eating a lot of salt may lower one’s blood pressure
